# High Neutrophil-to-Lymphocyte Ratio Predicts Hemorrhagic Transformation in Acute Ischemic Stroke Patients Treated with Intravenous Thrombolysis

**DOI:** 10.1155/2020/5980261

**Published:** 2020-02-27

**Authors:** Yong-Lin Liu, Jie-Kai Lu, Han-Peng Yin, Pei-Shan Xia, Dong-Hai Qiu, Man-Qiu Liang, Jian-Feng Qu, Yang-Kun Chen

**Affiliations:** ^1^Department of Neurology, Dongguan People's Hospital (Affiliated Dongguan Hospital, South Medical University), Dongguan, Guangdong Province, China; ^2^Department of Radiology, Dongguan People's Hospital (Affiliated Dongguan Hospital, South Medical University), Dongguan, Guangdong Province, China

## Abstract

**Background:**

The relationship between the neutrophil-to-lymphocyte ratio (NLR) and hemorrhagic transformation (HT) in acute ischemic stroke (AIS) treated with intravenous thrombolysis (IVT) remains unclear. This study assessed whether high NLR is associated with HT in this population.

**Methods:**

Data were prospectively collected for continuous patients with AIS treated with IVT and retrospectively analyzed. Clinical variables included age, sex, vascular risk factors, National Institutes of Health Stroke Scale (NIHSS) score, onset-to-treatment time, and initial hematologic and neuroimaging findings. HT was confirmed by imaging performed within 3 days after IVT. Symptomatic HT (sHT) was defined as NIHSS score increased by 4 points compared with that on admission according to previously published criteria. The NLR value was based on the blood examination before IVT, and high NLR was defined as ≥75th percentile.

**Results:**

The study included 285 patients (201 (70.5%) males, the mean age was 62.3 years (range 29–89)). Seventy-two (25.3%) patients presented with HT, including three (1.1%) with sHT. The median NLR was 2.700 (1.820–4.255, interquartile range). Seventy-one (24.9%) patients had a high NLR (≥4.255) on admission. Univariate analysis indicated that patients with HT had higher NIHSS scores (*P* < 0.001), systolic blood pressure (SBP), platelet counts, lymphocyte counts, and NLR (*P* < 0.001), systolic blood pressure (SBP), platelet counts, lymphocyte counts, and NLR (*P* < 0.001), systolic blood pressure (SBP), platelet counts, lymphocyte counts, and NLR (*P* < 0.001), systolic blood pressure (SBP), platelet counts, lymphocyte counts, and NLR (*P* < 0.001), systolic blood pressure (SBP), platelet counts, lymphocyte counts, and NLR (*P* < 0.001), systolic blood pressure (SBP), platelet counts, lymphocyte counts, and NLR (*P* < 0.001), systolic blood pressure (SBP), platelet counts, lymphocyte counts, and NLR (*P* < 0.001), systolic blood pressure (SBP), platelet counts, lymphocyte counts, and NLR (*P* < 0.001), systolic blood pressure (SBP), platelet counts, lymphocyte counts, and NLR (

**Conclusions:**

High NLR could be a useful marker for predicting HT in AIS patients after IVT.

## 1. Introduction

Intravenous thrombolysis (IVT) with recombinant tissue plasminogen activator (r-tPA) is an effective treatment for acute ischemic stroke (AIS) when administered within the hyperacute period [1, 2]. Hemorrhagic transformation (HT) is common in AIS with an incidence ranging from 8.5% to 40% [3–5], and symptomatic HT (sHT) is a risk factor for poor prognosis after AIS [6]. IVT has been reported to increase the incidence of HT markedly [7]. Atrial fibrillation (AF) [8, 9], National Institutes of Health Stroke Scale (NIHSS) score [10], blood glucose level [11], leukoaraiosis [12], dual antiplatelet agent treatment before IVT [13], and systolic blood pressure variability [14] are the predictors of HT after IVT.

In recent years, researchers have attempted to identify convenient serum biomarkers to help predict AIS outcomes. Several studies reported that a high neutrophil-to-lymphocyte ratio (NLR) was predictive of HT in AIS patients [15, 16]. However, there were limited data on the relationship between NLR and HT in AIS patients treated with IVT. The present study was performed to assess whether high NLR is associated with HT in AIS patients after IVT.

## 2. Methods

### 2.1. Patients

AIS patients treated with IVT after admission to Dongguan People's Hospital between 1 January 2016 and 31 May 2019 were continuously recruited. The inclusion criteria were as follows: (1) age >18 years, (2) AIS confirmed by magnetic resonance imaging (MRI), and (3) onset of stroke symptoms within 4.5 hours and treated with r-tPA. The exclusion criteria were as follows: (1) hemorrhagic lesions detected on initial computed tomography (CT), (2) temporary or permanent contraindications for MRI scan, (3) no acute lesion on diffusion-weighted imaging (DWI), and (4) additional endovascular therapy after IVT. This study was approved by the hospital ethics committee (approval number: KYKT2018-002). The consent of each subject was obtained in accordance with the Declaration of Helsinki.

### 2.2. Data Collection

NIHSS score, onset-to-treatment time (OTT), and blood pressure on admission were collected, as well as demographic data including age, sex, and history of hypertension, diabetes mellitus, smoking, AF, antiplatelet therapy, oral anticoagulant therapy, and previous stroke. Initial counts for white blood cells, neutrophils, lymphocytes, and platelets before IVT were also collected, and NLR was calculated accordingly.

### 2.3. MRI Analysis

As MRI is more sensitive than CT for detecting HT in AIS [17], we used MRI to confirm and categorize HT. A brain MRI scan was performed for each participant using a 3.0T system (Skyra, Siemens Medical Solutions, Erlangen, Germany) within 3 days after IVT. The sequences of MRI included T1-weighted imaging (T1WI), T2-weighted imaging (T2WI), DWI, and susceptibility-weighted imaging (SWI).

The parameters of each sequence were shown as follows: axial SE T1: time of repetition (TR) = 1500 ms, time of echo (TE) = 11 ms, field of view (FOV) = 220 mm, slice thickness/gap = 4 mm/1.2 mm, and time of acquisition = 1 min 26 s; Turbo spin echo (TSE) T2: TR = 4720 ms, TE = 96 ms, FOV = 220 mm, slice thickness/gap = 4 mm/1.2 mm, and time of acquisition = 1 min 50 s; DWI: TR = 4640 ms, TE = 67 ms, FOV = 230 mm, slice thickness/gap = 4 mm/1.2 mm, spin echo planar imaging (EPI) factor = 91, and acquisition time = 1 min 44 s; SWI: TR = 27 ms, TE = 20 ms, FOV = 220 mm, slice thickness/gap = 3 mm/0.6 mm, and time of acquisition = 2 min 28 s.

HT was defined as the secondary hemorrhage within or away from the infarction area, which appeared as hypointensive lesions on SWI [18, 19] and DWI [20]. Calcification was distinguished by CT combined with SWI. As chronic infarction lesions and corresponding old hemorrhage can be detected by T1-weighted imaging (T1WI) and T2-weighted imaging (T2WI), we used these sequences to differentiate acute HT from old hemorrhage.

1. sHT was defined as NIHSS score increase by ≥4 points compared with that on admission [21].
2. When HT was positive on MRI, the images were categorized into hemorrhagic infarct (HI) and parenchymatous hemorrhage (PH) according to the ECASS II criteria as follows: HI1, small petechiae along the margins of the infarct; HI2, confluent petechiae within the infarcted area but no space effect; PH1, blood clots in ≤30% of the infarcted area with some slight space-occupying effect; and PH2, blood clots in >30% of the infarcted area with substantial space-occupying effect.
3. Small vessel disease (SVD) burden was rated on brain MRI by the presence of lacunes, white matter hyperintensities, cerebral microbleeds, and perivascular spaces. The presence of each SVD feature was summed as an “SVD score” (range 0–4) [22].

Two neurologists (D.H.Q. and J.F.Q.) experienced in neuroimaging and trained by a neuroradiologist (M.Q.L.) evaluated the imaging findings for the presence of HT independently, blinded to the patients' clinical information. After observing the images individually, the two observers reviewed all the images to achieve final interobserver consensus.

### 2.4. Statistical Analysis

Statistical analyses were conducted using SPSS for Windows (v.20.0, IBM Corp., Armonk, NY, USA). Continuous variables with a normal distribution are reported as mean ± SD, and nonnormally distributed variables as median and interquartile range (IQR). All subjects were divided into two groups based on the presence of HT. Variables were compared using *t*-tests, Mann–Whitney *U* tests, Pearson *χ*^2^ tests, or Fisher's exact tests, as appropriate. Variables with *P* < 0.05 in the univariate analysis were included in further binary multivariate logistic regressions. Statistical significance was defined as *P* < 0.05 (two-sided).

## 3. Results

During the study period, 306 consecutive patients received IVT with r-tPA within 4.5 hours of stroke onset. In the present study, 21 patients were excluded for the following reasons: additional endovascular therapy after IVT (*n* = 2), permanent or temporary contraindication for MRI (*n* = 3), and no acute lesion found on DWI (*n* = 16). A total of 285 patients were ultimately included.

The average age of the 285 patients was 62.3 ± 12.0 years, and 201 (70.5%) patients were male. The median interval between stroke onset and MRI scanning was 42 (range, 13–65) hours. Antiplatelet agents (aspirin 100 mg/day or clopidogrel 75 mg/day) were prescribed 24 hours after IVT when PH (7 patients, 2.4%) was excluded, and no anticoagulants were prescribed during the acute phase. Among the 72 (25.3%) patients with HT confirmed by MRI, 44 (15.4%) presented with HI1, 21 (7.4%) with HI2, 4 (1.4%) with PH1, and 3 (1.1%) with PH2. Three (1.1%) patients had sHT. No remote HTs were found in our study. The mean OTT was 200.4 ± 55.9 minutes, and the median NIHSS score on admission was 7 (4–10, IQR). The median NLR was 2.700 (1.820–4.255, IQR). High NLR was defined as an NLR value ≥4.255 (75th percentile). Seventy-one (24.9%) patients had a high NLR before IVT. The demographic and clinical characteristics of this study are shown in Table 1.

### 3.1. Univariable Analysis

Compared with those without HT, patients with HT had significantly higher NIHSS scores (*P* < 0.001), systolic blood pressure (SBP), platelet counts, lymphocyte counts, and NLR (*P* < 0.05). They also had a greater prevalence of high NLR than those without HT (37.5% vs. 20.7% and *P*=0.004). Patients with HT were also more likely to have a history of hypertension and AF. The univariable analysis results are shown in Table 2.

### 3.2. Multivariate Logistic Regressions

Variables that were significantly different between the two groups in the univariable analysis were entered into subsequent logistic regression model. Since lymphocyte counts and high NLR were highly correlated (*r* = −0.499), we used two separate logistic regression models. Besides, hypertension and SBP on admission were also highly correlated (*r* = 0.412); therefore, SBP on admission was not included in the regression models to avoid the risk of multicollinearity. In model 1 (with high NLR), NIHSS score on admission (odds ratio (OR) = 1.110, 95% confidence interval (CI) = 1.015–1.044, and *P*=0.001), AF (OR = 3.986, 95% CI = 2.095–7.585, and *P* < 0.001), and high NLR (OR = 2.078, 95% CI = 1.078–4.003, *P*=0.029, sensitivity 0.375, and specificity 0.793) were significant predictors of HT. In model 2 (with lymphocyte counts), NIHSS score on admission (OR = 1.111, 95% CI = 1.050–1.175, and *P* < 0.001), AF (OR = 3.853, 95% CI = 2.048–7.248, and *P* < 0.001), and lymphocyte counts (OR = 0.522, 95% CI = 0.333–0.819, and *P*=0.005) were significantly related with HT. Platelet counts and hypertension were not significantly associated with HT in either model. The multivariate logistic regression results for HT risk factors are shown in Table 3.

## 4. Discussion

In our study, high NLR (≥4.255) was significantly associated with HT in AIS patients treated with IVT, which was in accordance with two previous studies [15, 16]. The mechanism of HT remains uncertain. The disruption of blood-brain barrier (BBB) and focal inflammation of the infarcted lesion have been reported to be correlated with HT [23]. In accordance with an existing report, neutrophils play a role in BBB in AIS [24]. Increased neutrophils can result in enhanced expression of matrix metalloproteinase-9 [25], which has been linked to BBB damage and HT in AIS patients [26–28]. Lymphocytes play important roles in inflammation [29, 30]. However, the precise effects depend on the subtype of lymphocytes. Some are neuroprotective [31, 32], while others exacerbate inflammation [33, 34]. A high NLR value represents high neutrophil counts and/or low lymphocyte counts. NLR is considered a good marker that simultaneously reflects the negative effects of neutrophils and positive effects of lymphocytes in stroke patients [35, 36]. High NLR was found to predict poor outcomes of AIS patients [37–39]. In our study, both lymphocyte counts and high NLR were significantly associated with HT in logistic regression analyses. However, absolute lymphocyte counts vary among individuals, even in healthy subjects. Thus, NLR may be a more stable and suitable marker than absolute lymphocyte counts for predicting HT. Neutrophil counts were not significant in the univariable analysis. However, since neutrophil counts were highly related with NLR (*r* = 0.676), our findings did not contradict previous studies. NLR is easily evaluated with a routine blood test, making it an economic and effective marker, even in regional hospitals.

There were several advantages to our study. First, to the best of our knowledge, it was one of the few that focused on the association between high NLR and HT in IVT-treated AIS patients. Second, all the participants had relatively complete data of neuroimaging including SWI. However, our results should be considered in the context of several limitations. First, repeated MR scanning was not performed, which might have led to underestimation of HT in the subacute phase. Second, we lacked dynamic NLR data, which would be considered a more effective predictor. Third, the numbers of cases with PH1, PH2, or sHT were too small to perform further analyses of these severe HT subtypes.

## 5. Conclusion

High NLR was a useful predictor of HT in AIS patients after IVT. Further prospective studies with larger sample sizes, repeated MR scans, and dynamic NLR are warranted.

## Figures and Tables

**Table 1:** Demographic and clinical characteristics of the study sample.

Characteristics	Mean (SD)/median (IQR)/*n* (%) (*n* = 285)
Age (years)	62.3 ± 12.0
Men (*n*, %)	201 (70.5%)
Hypertension (*n*, %)	211 (74.0%)
Diabetes mellitus (*n*, %)	79 (27.7%)
Smokers/ex-smokers (*n*, %)	102 (35.8%)
Atrial fibrillation (*n*, %)	67 (23.5%)
Previous stroke (*n*, %)	52 (18.2%)
PAT (*n*, %)	23 (8.1%)
POAT (*n*, %)	8 (2.8%)
Time of poststroke antiplatelet therapy	
Before MRI scan (*n*, %)	212 (74.4%)
After MRI scan (*n*, %)	66 (23.2%)
No antiplatelet therapy	7 (2.4%)
OTT (minutes)	200.4 ± 55.9
NIHSS score on admission	7 (4–10)^*∗*^
Platelet counts (10^9^/L)	214.0 ± 55.9
WBC counts (10^9^/L)	8.4 ± 2.9
Neutrophil counts (10^9^/L)	5.8 ± 2.8
Lymphocyte counts (10^9^/L)	1.9 ± 0.9
NLR	2.7 (1.8–4.3)^*∗*^
High NLR (*n*, %)	71 (24.9%)
Uric acid (mmol/L)	390.1 ± 108.6
BG on admission (mmol/L)	7.5 ± 3.2
SBP on admission (mmHg)	157.4 ± 24.8
DUB on admission (mmHg)	91.4 ± 16.5
SVD burden	1 (0–2)^*∗*^
Hemorrhagic transformation (*n*, %)	72 (25.3%)
HI1	44 (15.4%)
HI2	21 (7.4%)
PH1	4 (1.4)
PH2	3 (1.1%)
sHT (*n*, %)	3 (1.1%)

BG = blood glucose; DBP = diastolic blood pressure; HI = hemorrhagic infarct; NIHSS=National Institutes of Health Stroke Scale; NLR=neutrophil-to-lymphocyte ratio; OTT = onset-to-treatment time; PAT = previous antiplatelet therapy; PH = parenchymatous hemorrhage; POAT = previous oral anticoagulant therapy; SBP = systolic blood pressure; sHT = symptomatic hemorrhagic transformation; SVD = small vessel disease; WBC = white blood cell ^*∗*^median (25Q–75Q).

**Table 2:** Comparisons of clinicial and laboratory variables in IVT patients with and without HT.

Variable	With HT (*N* = 72)	Without HT (*N* = 213)	*t*/*X*^2^/*z* value	*P* value
Age^a^ (years)	64.0 ± 12.3	61.8 ± 11.9	−1.354	0.177
Men^b^	51 (70.8%)	150 (70.4%)	0.004	0.947
NIHSS score on admission^c^	10 (6–14.8)	6 (4–9)	−5.429	<0.001
Hypertension^b^	46 (63.9%)	165 (77.5%)	5.159	0.023
Diabetes^b^	19 (26.4%)	60 (28.2%)	0.085	0.770
Smokers^b^	25 (34.7%)	77 (36.2%)	0.048	0.827
AF^b^	35 (48.6%)	32 (15.0%)	33.759	<0.001
Previous stroke^b^	13 (18.1%)	39 (18.3%)	0.002	0.961
PAT^b^	3 (4.2%)	20 (9.4%)	1.979	0.213
POAT^b^	3 (4.2%)	5 (2.3%)	0.653	0.421
Antiplatelet therapy prescribed before MRI scan^b^	45 (62.5%)	167 (78.4%)	2.315	0.128
Uric acid^a^ (mmol/L)	369.2 ± 108.8	396.9 ± 107.9	1.844	0.066
OTT^a^ (minutes)	195.3 ± 52.6	202.2 ± 56.9	0.898	0.37
Platelet counts^a^ (10^9^/L)	201.3 ± 43.2	218.3 ± 59.0	2.246	0.025
WBC counts^a^ (10^9^/L)	8.6 ± 3.2	8.4 ± 2.8	−0.586	0.558
Neutrophil counts (10^9^/L)	6.3 ± 3.3	5.6 ± 2.6	−1.759	0.08
Lymphocyte counts (10^9^/L)	1.6 ± 0.7	2.1 ± 0.9	4.349	<0.001
High NLR^c^	27 (37.5%)	44 (20.7%)	8.16	0.004
BG on admission^a^ (mmol/L)	7.6 ± 3.4	7.5 ± 3.2	−0.280	0.779
SBP on admission^a^ (mmHg)	152.0 ± 24.1	159.2 ± 24.8	2.135	0.035
DBP on admission^a^ (mmHg)	90.1 ± 15.0	91.8 ± 17.0	0.754	0.452
SVD burden^c^	0 (0–1)	1 (0–2)	−1.434	0.152

^a^Mean (SD), *t*-test; ^b^*n* (%), chi-square test; ^c^Mann-Whitney *U* test. AF = atrial fibrillation; BG = blood glucose; DBP = diastolic blood pressure; HT = hemorrhagic transformation; NIHSS = National Institutes of Health Stroke Scale; NLR = neutrophil-to-lymphocyte ratio; OTT = onset-to-treatment time; PAT = previous antiplatelet therapy; POAT = previous oral anticoagulant therapy; SBP = systolic blood pressure; SVD = small vessel disease; WBC = white blood cell.

**Table 3:** Multivariate logistic regression of risk factors for hemorrhagic transformation.

Variable	*β*	OR (95% CI)	*P* value
Model 1 (with high NLR entered)			
NIHSS score on admission	0.100	1.110 (1.015–1.044)	0.001
Hypertension	−0.399	0.671 (0.352–1.280)	0.226
AF	1.383	3.986 (2.095–7.585)	<0.001
Platelet counts	−0.006	0.995 (0.988–1.001)	0.078
High NLR	0.731	2.078 (1.078–4.003)	0.029
Model 2 (with lymphocyte counts entered)			
NIHSS score on admission	0.105	1.111 (1.050–1.175)	<0.001
Hypertension	−0.440	0.644 (0.336–1.237)	0.181
AF	1.349	3.853 (2.048–7.248)	<0.001
Platelet counts	−0.004	0.996 (0.990–1.003)	0.240
Lymphocyte counts	0.650	0.522 (0.333–0.819)	0.005

AF = atrial fibrillation; NIHSS = National Institutes of Health Stroke Scale; NLR = neutrophil-to-lymphocyte ratio.

## Data Availability

The data used to support the findings of the study are available from the corresponding author upon request.
